# Motifs in the tau protein that control binding to microtubules and aggregation determine pathological effects

**DOI:** 10.1038/s41598-017-13786-2

**Published:** 2017-10-19

**Authors:** Aurélien Lathuilière, Pamela Valdés, Stéphanie Papin, Matthias Cacquevel, Catherine Maclachlan, Graham W. Knott, Andreas Muhs, Paolo Paganetti, Bernard L. Schneider

**Affiliations:** 1 Brain Mind Institute, Ecole Polytechnique Fédérale de Lausanne (EPFL), Lausanne, Switzerland; 2 AC Immune SA, Lausanne, Switzerland; 3 Centre of Interdisciplinary Electron Microscopy, EPFL, Switzerland; 4 Laboratory for Biomedical Neurosciences, Neurocenter of Southern Switzerland, Via Tesserete 46, CH-6900 Lugano, Switzerland

## Abstract

Tau pathology is associated with cognitive decline in Alzheimer’s disease, and missense tau mutations cause frontotemporal dementia. Hyperphosphorylation and misfolding of tau are considered critical steps leading to tauopathies. Here, we determine how motifs controlling conformational changes in the microtubule-binding domain determine tau pathology *in vivo*. Human tau was overexpressed in the adult mouse forebrain to compare variants carrying residues that modulate tau propensity to acquire a β-sheet conformation. The P301S mutation linked to frontotemporal dementia causes tau aggregation and rapidly progressing motor deficits. By comparison, wild-type tau becomes heavily hyperphosphorylated, and induces behavioral impairments that do not progress over time. However, the behavioral defects caused by wild-type tau can be suppressed when β-sheet breaking proline residues are introduced in the microtubule-binding domain of tau. This modification facilitates tau interaction with microtubules, as shown by lower levels of phosphorylation, and by the enhanced protective effects of mutated tau against the severing of the cytoskeleton in neurons exposed to vinblastine. Altogether, motifs that are critical for tau conformation determine interaction with microtubules and subsequent pathological modifications, including phosphorylation and aggregation.

## Introduction

Tau is a microtubule-associated protein (MAP), the main function of which is to stabilize microtubules implicated in axonal transport and axon structure^1^. In pathological conditions, and for reasons that remain to be elucidated, tau detaches from microtubules and assembles into paired helical filaments (PHF) to form intracellular inclusions^2,3^. In physiological conditions, tau is considered to be an unfolded protein mainly associated to microtubules. However, post-translational modifications, such as hyperphosphorylation and conformational changes, promote the transition towards pathological forms of tau. Several neurodegenerative diseases are characterized by the progressive accumulation of modified tau, including Alzheimer’s disease and frontotemporal dementia and FTDP-17^4^. In FTDP-17, several autosomal dominant missense mutations identified in the tau gene demonstrate that the modification of tau protein can cause neurodegeneration and dementia^5^. In Alzheimer’s disease, tau pathology is associated with its hyperphosphorylation, conformational changes and deposition in NFT, in the absence of any mutation in the tau gene. However, the development of the tau pathology correlates with neuronal loss and progressive cognitive deficits. This indicates that the post-translational modifications of normal tau confer a crucial role for this protein in neuronal dysfunction and neurodegeneration^6–8^.

In order to devise effective therapies, it is however important to identify critical steps in the gain of toxic functions of tau in neurons. Tau missorting, often associated with the compromised interaction of the protein with microtubules, is considered as an initial step in this process^9^. Several factors determine the affinity of different tau isoforms for microtubules, such as the number of repeats in the microtubule-binding domain (3 R versus 4 R), or the extent and site of tau phosphorylation. The phosphorylation-induced dissociation of tau from tubulin has been proposed to cause the loss of the microtubule-stabilizing function of tau, possibly contributing to neuronal degeneration^10–12^. When dissociated from microtubules, tau can then acquire various conformations, such as a compact “paperclip-like” conformation bringing together the N- and C-terminal portions of the protein^13^. Furthermore, the microtubule-binding domain contains hexapeptide motifs, which have a propensity to form β-sheets^14^. The transition to β-sheet structure is considered to be an important step, which promotes the formation of oligomers that can be competent for further aggregation into larger fibrillar structures such as PHF^15^. It is therefore important to determine how variations in the amino-acid sequence of the microtubule-binding domain critically control the kinetic of this process^16–18^.

To address this question, we used an *in vivo* model of tau pathology based on intracerebroventricular injections of AAV2/6 vectors in mouse neonates, to induce widespread overexpression of various forms of human tau in the forebrain. Tau pathology developed in the cortex and hippocampus, and induced motor behavioral deficits. To test the hypothesis that the propensity of the microtubule-binding domain to form β-sheets is a key factor in tau toxicity, we compared three variants of human 4R0N tau. The wild-type (WT) form was first compared to the pathogenic P301S mutant linked to FTDP-17. Conversely, to reduce the propensity of the microtubule-binding domain to form β-sheets, we introduced β-sheet-breaking proline residues in WT tau. The results show that the neuronal dysfunction caused by tau overexpression depends on the propensity of the microtubule-binding domain to form β-sheets. In addition, the observed behavioral defects correlate with differences in tau hyperphosphorylation and aggregation, as well as with the propensity of tau to protect neurites exposed to the microtubule-destabilizing agent vinblastine. These findings indicate that amino acid substitutions, or other modifications that may affect tau conformation in the microtubule-binding repeats, determine the progression and the type of tau-induced pathology in neuronal cells *in vivo*.

## Results

### AAV ICV injection in mouse neonates leads to overexpression of human tau in the forebrain

**Figure 1:**
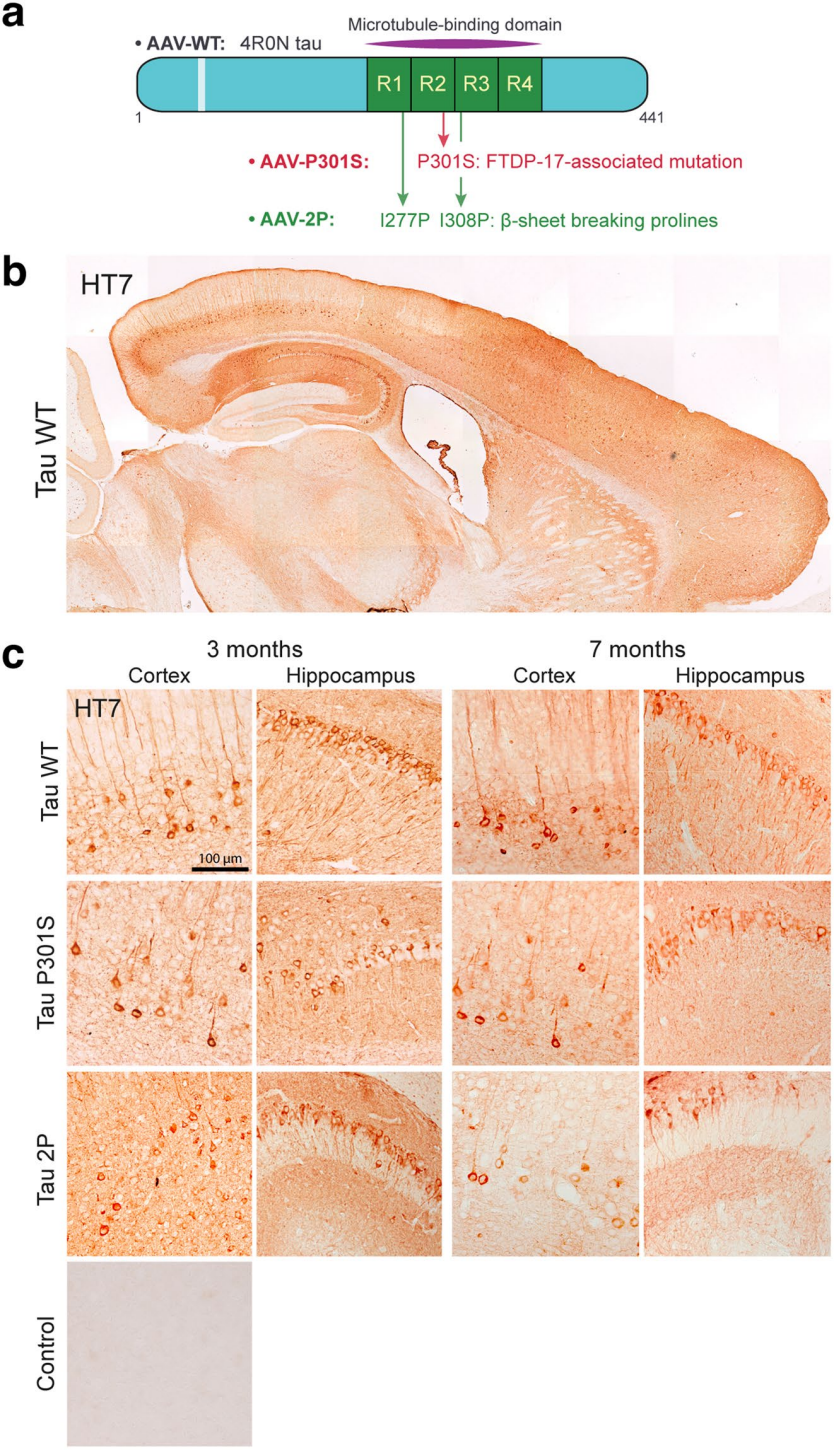
Intracerebroventricular injection of AAV-tau leads to widespread expression of human tau in the mouse forebrain. (**a**) Schema of the three tau-encoding sequences with amino acid substitutions. (**b**) Representative immunolabeling of human tau (HT7 antibody) in sagittal brain section, 3 months after injection of AAV-WT shows broad expression of human tau, mainly in cortex and hippocampus. (**c**) Representative HT7 immunolabeling in sagittal sections shows human tau-positive neurons in cortex and hippocampus (CA1) of mice injected with either AAV-WT, AAV-P301S or AAV-2P, 3 and 7 months after vector injection. Negative HT7 staining in a control mouse injected with AAV-maxFP is shown for comparison. Scale: 100 µm.

In order to model tau pathology in mice and explore behavioral changes induced by tau overexpression, we injected mouse neonates in the lateral ventricles (ICV injection, postnatal day 2) with AAV2/6-pgk vectors either encoding the WT form of human 4R0N tau (AAV-WT), or encoding the P301S mutant tau associated with frontotemporal dementia (AAV-P301S) (Fig. 1a). To modulate the propensity of tau to form β-sheets, we generated a vector encoding another form of the protein (2P tau) with amino acid substitutions in the domain that controls binding to microtubules (Fig. 1a). Two Ile-to-Pro mutations were introduced between R1 and R2 (I277P), and between R2 and R3 (I308P), two regions of the microtubule-binding domain that are critical for the transition towards a β-sheet conformation. These mutations have been previously reported to act as β-structure breakers in the highly aggregating ΔK280 mutant tau^19^. As a control group, mouse neonates were injected with the same dose of a vector encoding the fluorescent maxFP-Green protein (AAV-maxFP). This technique of AAV injection has been reported to efficiently transduce widespread regions of the mouse forebrain^20^. Indeed, at three months post-injection, overexpression of human tau was detected by immunohistochemistry on histological brain sections using an antibody specific for human tau (HT7). The human tau protein was broadly expressed in neuronal cells throughout the mouse forebrain (Fig. 1b), the highest expression being observed in the cortex and hippocampus (Fig. 1c). Expression was most prominent in large-sized neurons, such as pyramidal cells in cortical layers II/III and V, or hippocampal CA1 region (Fig. 1b and c). Regardless of the form of tau overexpressed (WT or P301S), the distribution of the protein was found to be both somatodendritic and axonal (Fig. 1c). As expected, there was no HT7 signal in mice injected with AAV-maxFP (Fig. 1c). Following ICV injection of AAV-2P, HT7 immunostaining showed similar expression of the tau protein in both the mouse cortex and hippocampus (Fig. 1c).

### Overexpression of human tau in the forebrain leads to motor behavioral impairments that can be prevented by 2P modification

**Figure 2:**
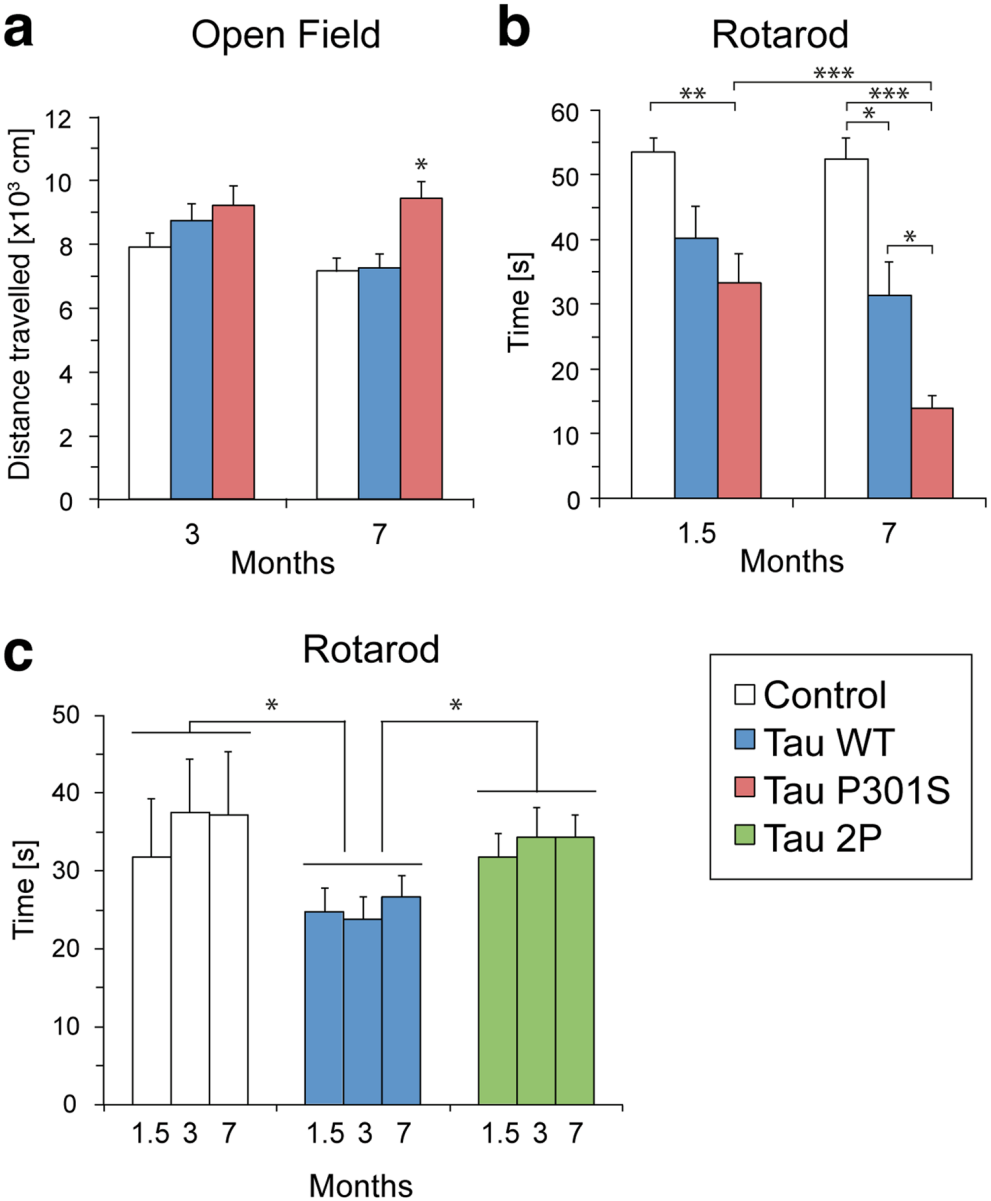
AAV-tau injection induces behavioral deficits: the effects of the P301S and 2P modifications. (**a**) Open field test performed at 3 and 7 months after AAV-tau injection, compared to the control group injected with AAV-maxFP. Data represent the distance travelled by each mouse on average, for each experimental group. (**b**) Rotarod test performed at 1.5 and 7 months after AAV-tau injection, compared to the control group injected with AAV-maxFP. The graph shows the average time on the rotating rod for each experimental group. AAV-maxFP: n = 15 mice; AAV-WT: n = 12; AAV-P301S: n = 13. Statistical analysis: two-way ANOVA (time × group) with repeated measures, followed by Newman-Keuls post-hoc test. Open field: group effect F(2,37) = 4.29; Rotarod: group × time effect F(2,37) = 4.38, *p < 0.05; **p < 0.01; ***p < 0.001. (**c**) Rotarod test to compare AAV-maxFP, AAV-WT and AAV-2P at 1.5, 3 and 7 months after vector injection. The graph shows the average time on the rotating rod for each experimental group. AAV-maxFP: n = 4 mice; AAV-WT: n = 16; AAV-P301S: n = 13. Statistical analysis: two-way ANOVA (time × group) with repeated measures, followed by Fisher’s LSD post-hoc test. Group effect F(2, 30) = 4.18, *p < 0.05.

Next, we sought to evaluate and compare the effect of each of these forms of tau on mouse behavior. As tau was highly expressed throughout the mouse cortex including the somatosensory and motor regions, we assessed exploratory locomotion and motor coordination, as such behavioral readouts are typically impaired in mouse models of tauopathies^21–23^. We first evaluated the exploratory behavior of the mice in the open field, at 3 and 7 months after vector injection (Fig. 2a). At three months, there was no significant difference between groups in the distance travelled by the mice. However, when the same test was performed with 7-months old mice, the group injected with the AAV-P301S vector showed a significant increase in travelled distance compared to both the control and AAV-WT groups (Fig. 2a). This effect may denote the progressive development of a hyperactive behavior in these mice, which is consistent with previous observations in mice injected with an AAV1 vector overexpressing the P301L tau mutant^24,25^.

We also explored the impact of tau overexpression on motor performance using the rotarod test. Remarkably, already at 1.5 months after injection the AAV-P301S group showed a significant decrease in latency to fall from the rod rotating at 35 rpm when compared to the AAV-maxFP control group (Fig. 2b). At 7 months, the effect of tau was even more pronounced, as the latency to fall was 14.0 ± 1.8 sec in the AAV-P301S group, and 31.3 ± 5.2 sec in the AAV-WT group, both significantly different from the control group (52.5 ± 3.2 sec). In particular, expression of P301S tau worsened the motor performance of the mice, which was found to be significantly lower than the two other groups (Fig. 2b). Whereas the deficits caused by WT tau remain stable, the P301S mutant tau leads to a time-dependent deterioration of the motor phenotype, indicating a progressive pathology.

In a second experiment, we compared the effects of AAV-WT and AAV-2P on mouse behavior using the rotarod test (Fig. 2c). Again, as compared to the AAV-maxFP, AAV-WT induced a significant decrease in the latency to fall already at 1.5 months post-injection, and which remained stable at 3 and 7 months after vector injection (Fig. 2c). Remarkably, however, the presence of the two β-structure breaking prolines in tau (AAV-2P) reversed this effect, as the mice injected with the AAV-2P vector performed as well as the control AAV-maxFP mice in the rotarod test (Fig. 2c). Over the whole course of the experiment, their latency to fall was significantly increased for the AAV-2P mice compared to the group injected with AAV-WT (Fig. 2c).

### P301S tau progressively accumulates in the forebrain following ICV AAV injection in neonatal mice

Next, in order to correlate the observed behavioral effects with possible alterations of the tau protein, we performed a biochemical characterization of human tau in AAV-WT and AAV-P301S mice. The expression level of tau in the mouse forebrain was assessed at different ages by western blotting in total brain homogenates. Using an antibody that recognizes both mouse and human tau (tau5), we observed an increase in the expression of a tau species with an apparent molecular weight of approximately 55 kDa, which indeed corresponds to 4R0N human tau (Supplementary Fig. S1). This isoform is also abundantly present in protein extracts from the neocortex of an Alzheimer’s patient and of an unaffected control. Compared to the level of endogenous tau in the forebrain of a mouse injected with the control AAV-maxFP vector, the amounts of total tau found in the AAV-WT and AAV-P301S mice were nearly doubled.

**Figure 3:**
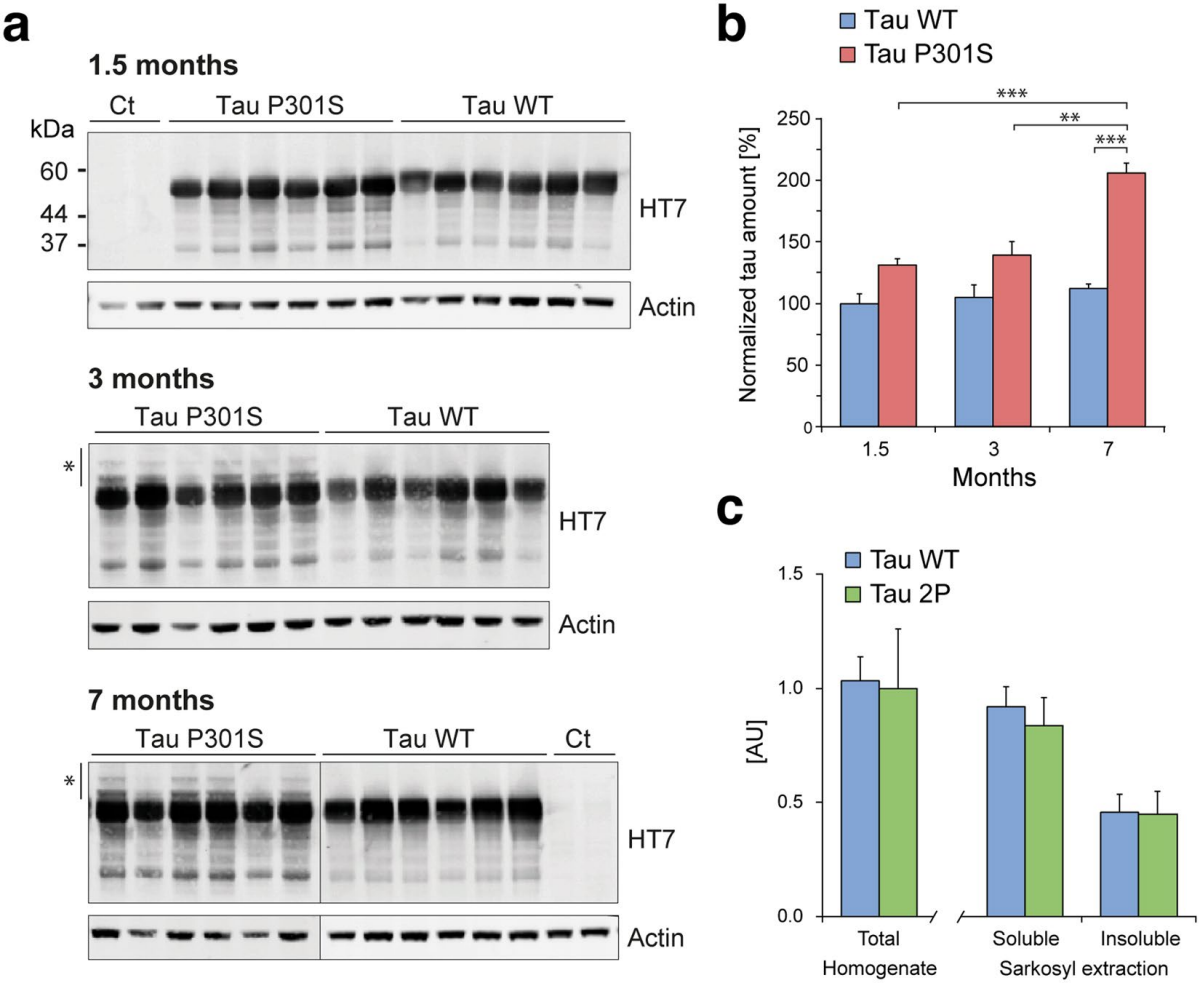
The level of human tau increases over time in the brain of mice injected with AAV-P301S. (**a**) Detection of overexpressed tau using the human tau-specific HT7 antibody in mouse forebrain homogenates at 1.5, 3 and 7 months post-AAV injection. Actin is used as loading control. *Indicates higher molecular weight human tau species observed in the P301S group of mice. (**b**) Relative human tau levels measured by western blotting of mouse forebrain homogenates (1.5, 3 and 7 months post- injection). Blots are shown in Supplementary Figure 1. For each animal, human tau values are normalized to actin and plotted as percentage of the mean value for the AAV-WT group at 1.5 months. Statistical analysis: two-way ANOVA followed by Tukey’s post-hoc test, group × time effect F(2,30) = 4.89, **p < 0.01; ***p < 0.001. (**c**) The graph compares the level of human tau (HT7/TAU-13 AlphaLisa) in total soluble proteins from forebrain homogenates, as well as in the soluble and insoluble fractions following sarkosyl extraction. Samples are derived from AAV-WT and AAV-2P mice, 7 months after AAV injection; n = 8 mice per group.

For a more specific analysis of overexpressed tau between the WT and P301S mice, we used the HT7 antibody that specifically detects human tau. As shown in Fig. 3a and b, the amount of human tau in brain protein homogenates appeared stable up to 7 months post-injection in the AAV-WT group of mice. In contrast, in mice injected with the vector encoding P301S mutant tau, we observed a time-dependent accumulation of human tau whereby P301S tau was significantly increased at 7 months when compared to 1.5 and 3 months (Fig. 3a,b). P301S tau reached nearly a two-fold higher level than WT tau in 7-months old mice. Notably, we noticed the presence of high-molecular weight species of the tau protein in brain extracts of the AAV-P301S mice (Fig. 3a).

In the experiment comparing AAV-WT and AAV-2P, the amount of soluble human tau present in forebrain homogenates was similar in these two groups of mice, at 7 months after vector injection (Fig. 3c). Moreover, a sarkosyl extraction demonstrated similar levels of human tau in both the sarkosyl-soluble and insoluble fractions, indicating that the 2P modification did not have any effect neither on the overall tau expression nor on the deposition of insoluble human tau (Fig. 3c).

Altogether, these results showed that injection of AAV-tau vectors leads to sustained levels of human tau in the mouse forebrain, which are comparable across the different forms of tau. However, the P301S mutant form of tau tends to accumulate over time. Next, we further analyzed tau misfolding and phosphorylation as a function of time, as these modifications are associated with tauopathies.

### Aggregated forms of tau accumulate more rapidly in mice injected with the AAV vector expressing the P301S tau mutant

**Figure 4:**
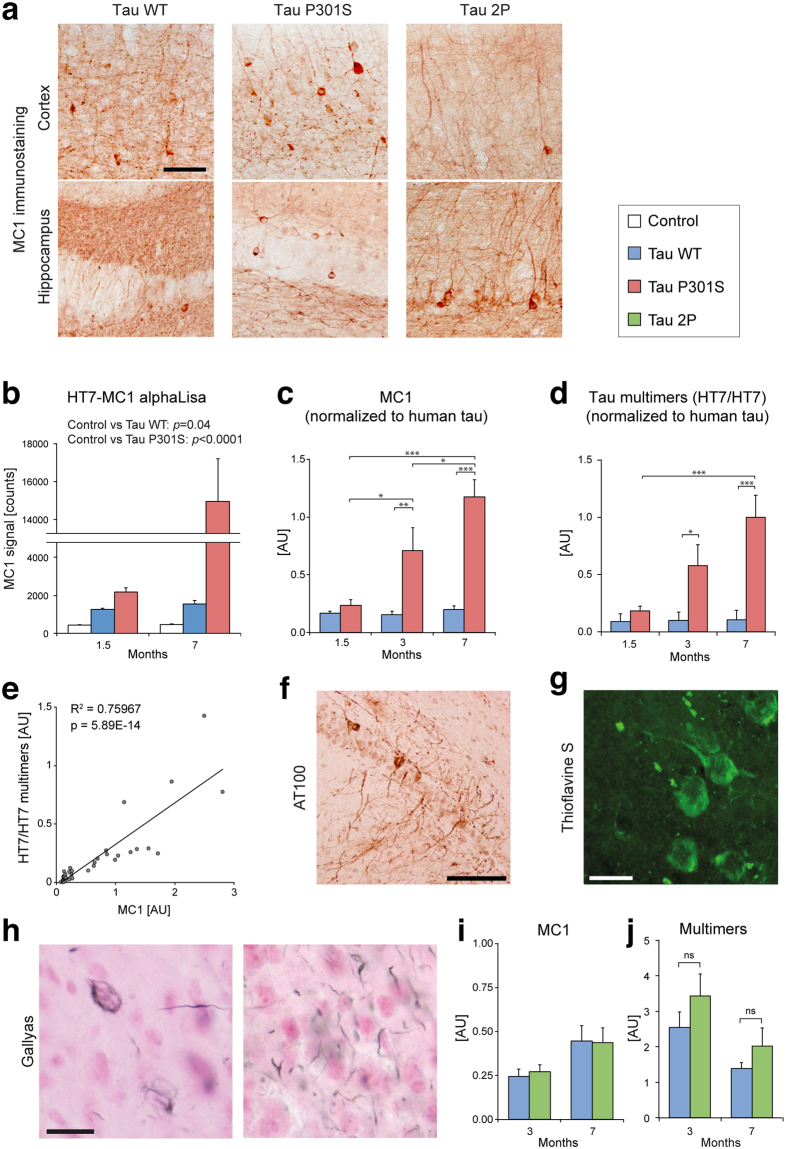
Aggregated forms of human tau progressively accumulate in the brain of mice injected with AAV-P301S. (**a**) Representative images of the hippocampus immunolabeled with the conformational MC1 antibody. Note the presence of MC1-positive neurons and neurites, 7 months after injection of either AAV-WT, AAV-P301S or AAV-2P. Scale bar: 100 μm. (**b**) Quantification of MC1-positive misfolded tau in forebrain protein homogenates using an HT7/MC1 AlphaLisa at 1.5 and 7 months post-injection. Statistical analysis: Kruskal-Wallis test, H(2, N = 38) = 28.25. (**c**) HT7/MC1 values are normalized for total human tau determined with a HT7/TAU-13 AlphaLisa. (**d**) The amount of human tau multimers is determined with a HT7/HT7 single-epitope AlphaLisa and normalized for total human tau. For c and d: data represent relative arbitrary units. Statistical analysis: two-way ANOVA followed by Tukey’s post-hoc test, group × time effect (**c**) F(2, 36) = 6.55, (**d**) F(2, 36) = 3.88, *p < 0.05; **p < 0.01; ***p < 0.001. (**e**) Significant correlation between the amount of MC1-immunoreactive human tau and human tau multimers (Pearson). (**b**–**d**) 1.5 and 3 months: AAV-maxFP: n = 2; AAV-WT and AAV-P301S: n = 6. 7 months: AAV-maxFP: n = 6; AAV-WT: n = 8; AAV-P301S: n = 10 mice. (**f**–**h**) Representative histological analysis, 7 months after AAV-P301S injection: (**f**) AT100 immunolabeling of phosphorylated tau in the hippocampus (CA1). Scale bar: 100 µm. (**g**) Thioflavine-S positive neurofibrillary deposits in cortical neurons. Scale bar: 25 µm. (**h**) Gallyas silver staining of argyrophilic filamentous inclusions in cortical neurons. Scale bar: 25 μm. (**i**) Detection of misfolded tau (HT7/MC1 AlphaLisa) in mouse forebrain homogenates, at 3 and 7 months after either AAV-WT or AAV-2P injection. Single mouse values were normalized for total human tau (HT7/TAU-13 AlphaLisa); n = 8 mice per group. (**j**) Detection of tau multimers (HT7/HT7 single-epitope AlphaLisa); n = 8 mice per group. Statistical analysis: two-tailed Student’s t test.

Conformational changes in the tau protein, including transition towards a “paperclip-like” conformation, or towards the formation of β-structures aggregating into PHF, occur in the development of tauopathies^26^, and can precede the formation of NFTs^27^. We analyzed the presence of misfolded tau in the brain of AAV-injected mice using conformation-specific tau markers. Immunohistochemistry for MC1, an antibody specific for early changes in tau conformation, revealed the presence of abnormally folded tau in the brain regions overexpressing human tau, including the cortex and hippocampus (Fig. 4a). The staining was observed in the AAV-WT, AAV-P301S and in the AAV-2P groups of mice, 7 months after vector injection.

To quantitatively assess tau misfolding and multimerization, we first developed three AlphaLisa assays to analyze tau in protein homogenates from the mouse forebrain. The assays were conceived using the human-specific HT7 monoclonal antibody as capture antibody. For the first assay, we used MC1 as reporter antibody for early conformational changes of tau (Fig. 4b,c). Already at 1.5 months after injection, we detected a significantly higher MC1 signal in the mice injected with the tau-expressing vectors as compared with mice injected with the control vector (Fig. 4b). These data indicate that human tau protein rapidly acquires a pathology-prone conformation when overexpressed in the mouse forebrain. In order to effectively compare the misfolding of tau between animals, the signal obtained with the HT7/MC1 assay was normalized to the total amount of human tau, determined for each sample in a parallel AlphaLisa with the HT7/TAU-13 monoclonal antibody pair. As shown in Fig. 4c, the normalized MC1-positive tau remained constant over time in the AAV-WT mice. In contrast, the injection of the AAV-P301S vector induced a progressive increase in the amount of misfolded MC1-tau, which was increased by 4.6- and 5.9-fold compared to AAV-WT at 3 and 7 months post-injection, respectively.

Tau multimers were measured using the same HT7 antibody for capture and detection in a single-epitope immunodetection design that cannot detect monomeric tau forms (Fig. 4d). When measuring HT7/HT7 tau multimers, we made very similar observations (compare Fig. 4c with d), highly correlated with the levels of the MC1 signal (Fig. 4d,e). Again, while aggregated tau remained stable in the AAV-WT group of mice, a progressive accumulation of tau multimers occurred in the forebrain of the AAV-P301S group. Compared to WT tau, the level of P301S tau multimers was increased by 3.1- and 3.3-fold at 3 and 7 months post-injection, respectively. Overall, we find a prominent effect of the P301S mutation on the misfolding and aggregation of the tau protein *in vivo*.

Biochemical detection of aggregated P301S tau indicates that fibrillar tau may accumulate over time in these animals. Therefore, we performed additional histological analyses using markers for more advanced tau pathology. The AT100 antibody was used to detect abnormal tau phosphorylation on residues Thr 212/Ser 214, also present on PHF (Fig. 4f)^28^. To further substantiate the presence of tau deposition, we used the thioflavine-S and Gallyas silver stainings, which reveal neurofibrillary deposits of PHF. We observed the presence of AT100-positive neuronal cell bodies and processes only in the group of mice injected with the AAV-P301S vector (Fig. 4f). These neurons were mainly distributed in the cortex and hippocampus 7 months after vector injection. In the same samples, we also observed the presence of neurons positive for thioflavine-S in the cortex (Fig. 4g), as well as the deposition of argyrophilic filamentous inclusions stained with Gallyas (Fig. 4h). The filamentous inclusions were found in the neuronal soma or appeared as structures resembling neuropil threads (Fig. 4h). Of note, we did not detect any sign of advanced tau pathology in any of the mice injected with AAV-WT. Altogether, these data confirmed the progressive modification of tau leading to misfolding and aggregation, which was most evident for the P301S pathogenic mutation of tau.

When comparing WT and 2P tau, we did not find any significant effect of the 2P modification neither on the amount of misfolded tau in the mouse forebrain (HT7/MC1 AlphaLisa normalized to total human tau, Fig. 4i), nor on the amount of tau multimers (HT7/HT7 AlphaLisa normalized to total human tau, Fig. 4j), at 3 and 7 months post-injection.

### WT tau becomes more phosphorylated than P301S tau on residues Ser 396, Ser 202/Thr 205, Thr 181 and Thr 231 following overexpression in the mouse forebrain

**Figure 5:**
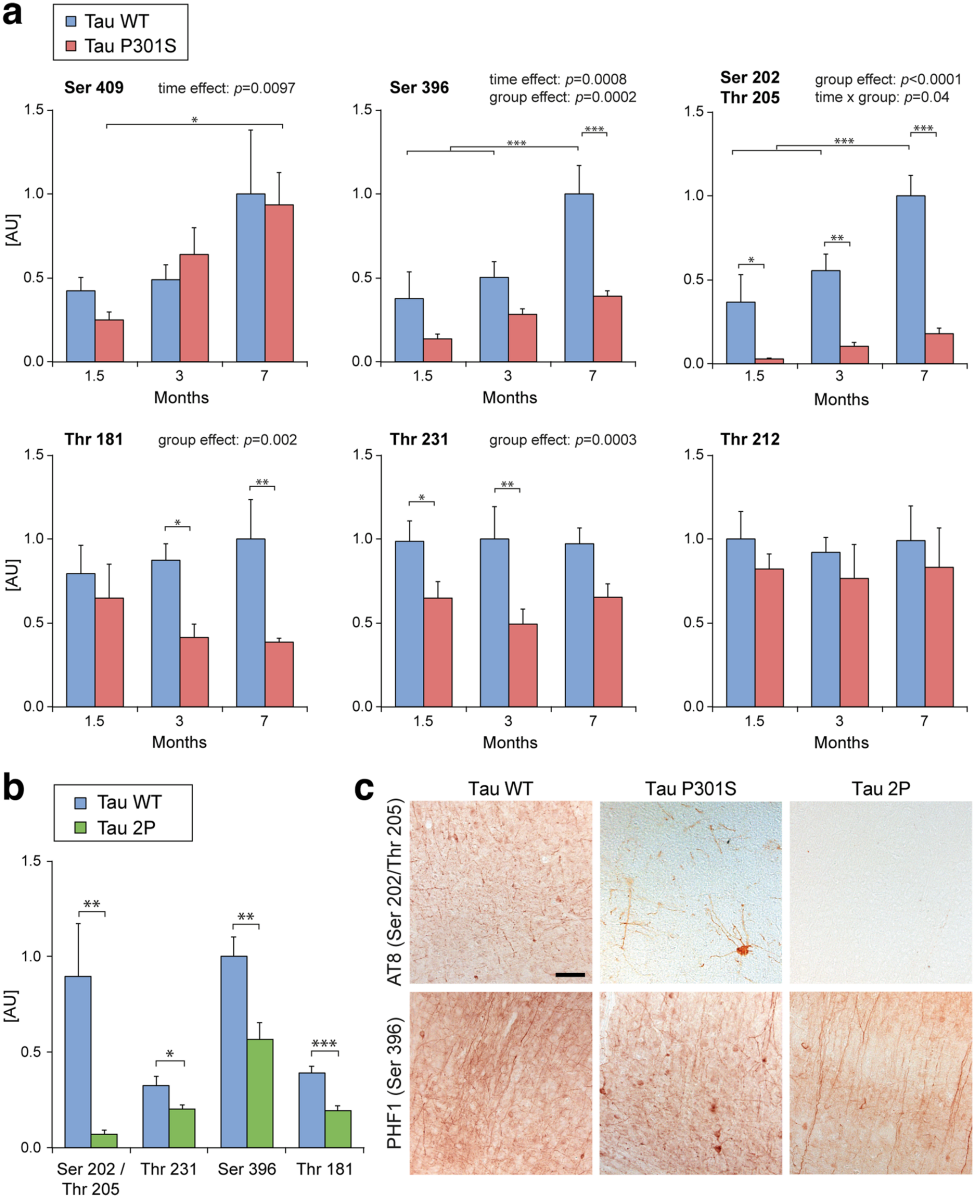
Human WT tau is more subjected to phosphorylation in the mouse forebrain than P301S and 2P tau. (**a**) AlphaLisa assays are performed on forebrain protein homogenates to determine the levels of human tau phosphorylation at the indicated residues, as function of age (1.5, 3 and 7 months after injection) and tau variant (AAV-WT or AAV-P301S). Data represent relative levels of phosphorylated tau, normalized to the total amount of human tau determined in a parallel AlphaLisa. Note that WT tau is more heavily phosphorylated than P301S tau at residues Ser396, Ser 202/Thr 205, Thr 181 and Thr 231. For all conditions: n = 6. Statistical analysis: two-way ANOVA followed by Tukey’s post-hoc test, *p < 0.05; **p < 0.01; ***p < 0.001. (**b**) Representative images of phosphorylated tau deposition, 7 months after injection of AAV-WT and AAV-P301S. Immunolabeling with anti-phospho tau antibodies AT8 (Ser202/Thr205) in the cortex (left panels) and with PHF1 (Ser396) in the hippocampus (right panels) are shown. Note the localization of P301S tau in the neuronal soma and dendrites. Scale bar: 100 μm. (**c**) AlphaLisa quantification of human tau phosphorylation at residues Ser 202/Thr 205, Thr 231, Ser 396 and Thr 181 in mouse forebrain homogenates, 7 months after injection of either AAV-WT or AAV-2P. The graphs represent the levels of phosphorylated tau relative to total human tau; n = 8 mice per group. Statistical analysis: two-tailed Student’s t test. *p < 0.05; **p < 0.01; ***p < 0.001. (**d**) Representative immunodetection of phosphorylated tau using the AT8 (Ser 202/Thr 205) and PHF1 (Ser 396/Ser 404) antibodies in sagittal sections of the mouse cortex 7 months after injection of either AAV-WT or AAV-2P. Note the decreased immunoreactivity for phosphorylated tau in AAV-2P-injected mice. Scale bar: 100 μm.

The deposition of abnormal tau hyperphosphorylated on several residues is a typical feature of several tauopathies. The phosphorylation pattern of tau was therefore assessed in the brain homogenates obtained from the mice at different ages. For quantitative determination of tau phosphorylation at specific sites associated with tauopathies^29^, several additional AlphaLisa assays were established. As before, we used the HT7 or TAU-13 capture antibodies coupled to the donor beads, whereas detection was performed with monoclonal antibodies specific to different phosphorylated tau residues coupled to the acceptor beads. For each sample, the abundance of the phosphorylated epitopes was normalized to the total amount of human tau, in order to also take into account the different levels of WT and P301S tau as a function of age. Overall, the data demonstrated tau phosphorylation as early as 1.5 months after vector injection in both the AAV-WT and AAV-P301S groups. However, phosphorylation extent varied for most residues, as a function of age and presence of the P301S mutation (Fig. 5a).

The level of phosphorylation of human tau at residues Ser 409, Ser 396 and Ser 202/Thr 205 was dependent on the age of the animals, progressively increasing between 1.5 months and 7 months post-injection (Fig. 5a). Remarkably, tau phosphorylation on residues Ser 396, Ser 202/Thr 205, Thr 181 and Thr 231 was more abundant on WT tau than on P301S tau (Fig. 5a). In 7 month-old mice, this effect was particularly evident on the phosphorylation of the Ser 396, Ser 202/Thr 205 and Thr 181 residues. Therefore, although P301S tau accumulates as a function of age, the relative contribution of phosphorylation on certain residues is more pronounced for WT tau. In contrast, age and mutation effects were different for other phosphorylation sites. The level of phosphorylation of Ser 409 and Thr 212 was nearly identical for WT and P301S tau (Fig. 5a). In particular, phosphorylation of Thr 212 showed no dependency on the presence of tau mutation and age.

In addition to these biochemical assays, the presence of hyperphosphorylated tau was investigated by immunohistology using two phospho-tau specific antibodies: AT8 (phosphorylated Ser 202/Thr 205) and PHF1 (phosphorylated Ser 396/Ser 404). In 7-month-old mice, a strong immunoreactivity for AT8 and PHF1 antibodies was found in the cortex and hippocampus, in the AAV-WT group and to a lesser extent in the AAV-P301S group (Fig. 5b). Whereas immunoreactivity for phospho-tau epitopes was mainly observed in the axons of neurons overexpressing WT tau, phosphorylated P301S was more evident in the somatodendritic compartment. Altogether, these results indicate that overexpression of WT tau is characterized by a more extensive hyperphosphorylation pattern, which tends to further develop over time when compared to P301S tau.

### 2P-modified tau is less hyperphosphorylated than wild-type tau

Next, we compared the phosphorylation status of WT and 2P tau. The level of phosphorylation was assessed on sites Ser 202/Thr 205, Thr 231, Ser 396 and Thr 181, which were found highly phosphorylated on WT tau (see Fig. 5a). In contrast to the absence of effects for misfolding and aggregation, the 2P modification clearly affected the pattern of tau hyperphosphorylation. When compared to WT tau, phosphorylation was significantly decreased in the 2P tau group for all the residues analyzed, 7 months after vector injection (Fig. 5b,c). The effect of the 2P modification was particularly dramatic on the phosphorylation of Ser 202/Thr 205, which was almost completely suppressed (Fig. 5c). Moreover, histological analysis confirmed these findings. Indeed, the immunoreactivity for AT8 (phosphorylation at Ser 202/Thr 205) was nearly absent, whereas PHF1 immunoreactivity (phosphorylation at Ser 396/Ser 404) was partly decreased in the cortex of the mice overexpressing 2P tau, as compared to WT tau (Fig. 5b). Overall, the main effect of the 2P modification appears to be a significant decrease in the extent of human tau hyperphosphorylation.

Altogether, the comparison between WT and 2P tau suggests that the behavioral impairments caused by the overexpression of human WT tau are likely to be conferred by increased burden of hyperphosphorylation, rather than by the accumulation of misfolded tau protein. Remarkably, amino acid substitutions that affect the conformation of the microtubule-binding region of 4R0N tau have major effects on the downstream accumulation of hyperphosphorylated tau, and prevent the impairments of motor behavior observed in this mouse model.

### Ultrastructural analysis of the mouse cortex reveals microtubule bundling in neurons overexpressing P301S and 2P-modified tau

The phosphorylation of tau affects the association of the protein to microtubules. To determine if the observed differences in the phosphorylation status of overexpressed tau correlate with changes in microtubule stability, we used transmission electron microscopy (TEM) to explore possible ultrastructural changes in the mouse neocortex.

**Figure 6:**
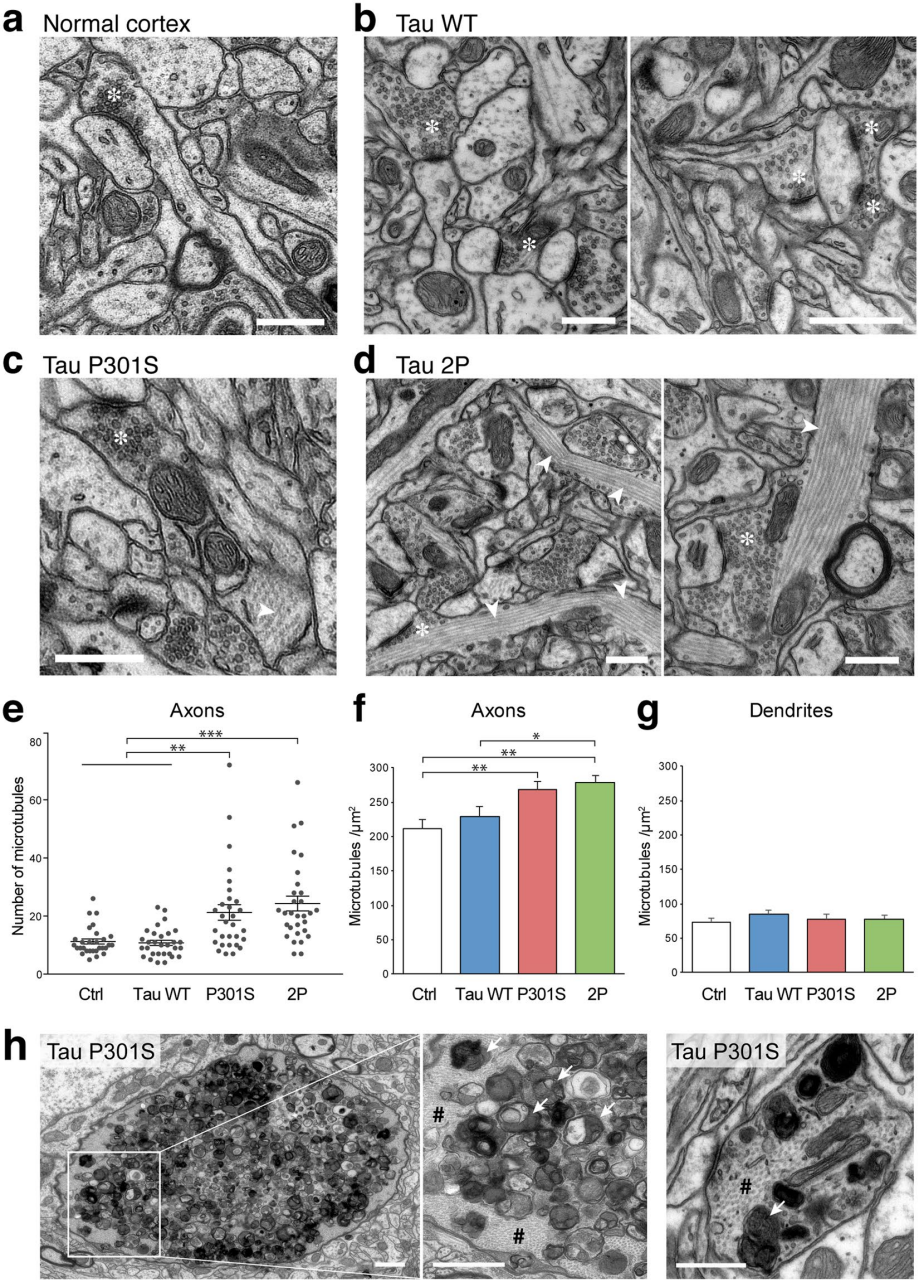
2P-modified and P301S tau increase microtubule number and density in axonal profiles in the mouse cortex. Representative electron micrographs from layer II/III of mouse neocortex neuropil, 7 months post-vector injection. Images are taken close to the site of injection. (**a**,**b**) In the mouse injected with AAV-WT, the neuropil shows normal morphology with microtubules scattered in axon terminals and dendrites, similar to the ultrastructure observed in a non-injected mouse (normal cortex). (**c**,**d**) In mice injected with either AAV-P301S or AAV-2P, there is an abundance of cytoskeletal elements, with high densities of aligned microtubules (indicated by white arrowheads) located in axons. These are also seen in the axonal boutons (indicated by *). Scale bars: 500 nm. (**e**) Quantification of the number of microtubules per axon in each condition. (**f**,**g**) Quantification of microtubule density in individual axons. Note the significant increase in microtubule number and density in the AAV-P301S and AAV-2P conditions. Statistical analysis: one-way ANOVA followed by Tukey’s post-hoc test, n = 29–30 axons per condition, *p < 0.05; **p < 0.01; ***p < 0.001. (**h**) Representative electron micrographs from the neocortex, 7 months post-injection of the AAV-P301S tau vector. Enlarged axons containing high densities of aligned microtubules (indicated by #) are present, as well as axonal swellings containing large accumulations of degenerated organelles including whorls, membrane inclusions and degenerating mitochondrial profiles (white arrows). Scale bars: 1 µm (left panels) and 500 nm (right panel).

Following injection of the WT tau-encoding vector, microtubule arrangement appeared normal and indistinguishable from the normal situation (Fig. 6a,b). However, in both the P301S and 2P tau groups, local increases in the density of the microtubule network were observed mainly in axons (Fig. 6c,d). This includes the axonal boutons, where the microtubules could also be seen close to the synaptic vesicles, and active zone (Fig. 6d). Microtubules were often organized in bundles with hexagonal symmetry, similar to the tau-induced microtubule fascicles described previously^30^. To quantify this effect, the number of microtubules was determined in individual axons. In both the P301S and 2P conditions, the number of microtubules per axon was significantly increased compared to either non-injected or AAV-WT injected mice (Fig. 6e). When the number of microtubules was normalized to the cross-sectional area of the axon, we also observed a significant increase in density in the P301S and 2P mice versus the control non-injected mice (Fig. 6f). In contrast, the microtubule density was not changed in the dendrites (Fig. 6g). Therefore, we find that the P301S and 2P variants of tau lead to clear changes in microtubule number and density. These results suggest that these two forms of tau, which have a modified amino-acid sequence in the microtubule-binding domain, are more likely to interact with tubulin than WT tau, which is consistent with the reduced level of tau phosphorylation observed in these conditions.

Ultrastructural analysis of the mouse cortex by TEM also revealed typical features of degenerating neurons. In particular, in the AAV-P301S mouse, we noticed axonal structures containing large abnormal accumulation of defective organelles or autophagic material at 7 months post-injection (Fig. 6h). For histological examination of neurodegeneration in the mouse forebrain, cresyl violet staining was used to reveal neuronal cell bodies in sagittal brain sections. In the animals injected with AAV vectors encoding either WT tau (4 out of 6 animals) or P301S tau (all the animals), there was an evident thinning of the cortical regions near the site of vector injection (Supplementary Fig. S2a). The hippocampus remained mostly intact. Degeneration of the mouse cortex was already observed at 1.5 and 3 months after vector injection. In contrast, there were no apparent morphological changes in the mice injected with the control AAV-maxFP vector at month 3, indicating that this effect was mainly caused by the human tau protein and not by mechanical injury (Supplementary Fig. S2a). In the AAV-2P injected group, only one out five mice showed a noticeable degeneration of the cortex. In the mice from the AAV-WT and AAV-P301S groups which displayed cortical neurodegeneration, it appeared that the most superficial layers of the cortex (layer I-V) were the most affected, as shown with a NeuN immunostaining to reveal cortical layers (Supplementary Fig. S2b).

### Overexpression of 2P tau inhibits the effects of vinblastine on microtubules in cortical neurons

**Figure 7:**
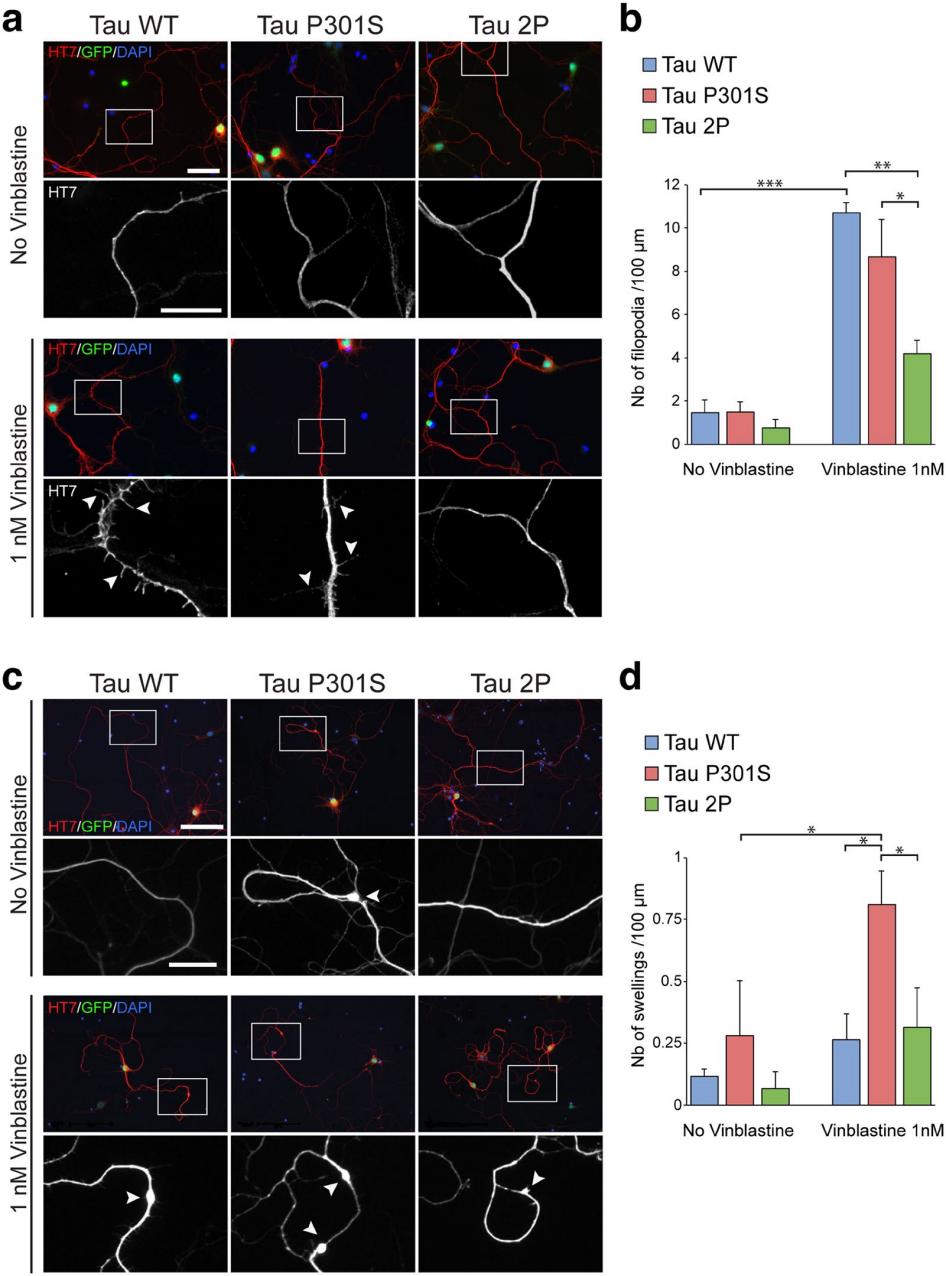
2P-modified tau has protective effects on the microtubule network in primary cortical neurons exposed to vinblastine. Overexpression of Tau 2P prevents the formation of filopodia in mouse cortical neurons exposed to vinblastine. (**a**) Immunodetection of human tau (HT7) and GFP in cortical neurons (DIV5) infected with a bicistronic AAV-GFP/tau vector to overexpress each of the different human tau variants (WT, P301S or 2P). Nuclear expression of GFP is used to identify the transduced neurons. Upper panels show representative images of neurons in the control condition. Lower panels show cortical neurons after 24 h exposure to 1 nM vinblastine. Arrowheads indicate the presence of filopodia on the neuronal processes. Scale bars: 40 µm and 20 µm (lower panels). (**b**) Quantification of the number of filopodia normalized to neurite length. Exposure to 1 nM vinblastine increases the number of filopodia. Note that the overexpression of 2P tau significantly reduces the number of neuritic filopodia compared to WT and P301S tau. (**c**) Seven days exposure to 1 nM vinblastine leads to the formation of neuritic swellings. Experimental conditions and immunodetection are similar to panel (a). Lower panels show higher magnification of HT7 immunostaining in the neurites of neurons overexpressing human tau. Arrowheads indicate the presence of neuritic swellings. Scale bars: 100 µm and 20 µm (lower panels). (**d**) Quantification of the number of swellings normalized to neurite length. Exposure to 1 nM vinblastine increases the number of swellings in neurons overexpressing P301S tau. For all conditions: n = 3. Statistical analysis: two-way ANOVA followed by Tukey’s (**b**) or Fisher’s LSD (**d**) post-hoc tests, *p < 0.05; **p < 0.01; ***p < 0.001.

Next, we sought to further determine the effects of the different tau variants on microtubules in the neurites of primary cortical neurons *in vitro*. To assess the protective effects of tau on microtubules, we induced defects by exposing the neurons to vinblastine, which interacts with tubulin heterodimers in the same location as tau and competes with tau binding^31^. When cultures of mouse cortical neurons overexpressing tau were exposed to 1 nM vinblastine for 24 h, we observed a dramatic increase in the number of neuritic filipodia, a clear indicator of microtubule severing (Fig. 7a and b). We did not observe any significant difference in the number of primary or secondary neuritic branches.

In neurons transduced with AAV-tau, HT7 immunostaining was used to reveal the neuronal processes expressing human tau, and determine the frequency of filopodial protrusions in neurons expressing each of the different tau variants. A similar number of filopodia was observed in neurons overexpressing WT and P301S tau (Fig. 7a and b). Remarkably, the effect of vinblastine on the formation of filopodia was significantly diminished in neurons overexpressing 2P tau, compared to both WT and P301S tau. In neurons overexpressing 2P tau, the number of filopodia was not significantly different from the control neurons not exposed to vinblastine. This result indicates that this form of tau has enhanced protective effects against microtubule destabilization caused by vinblastine.

Following longer exposure to vinblastine (7 days), we also observed the formation of neuritic swellings in tau-overexpressing cortical neurons, and quantified this effect (Fig. 7c and d). The number of these neuritic swellings was significantly increased in neurons overexpressing P301S tau when treated with 1 nM vinblastine, as compared to neurons expressing either WT or 2P tau (Fig. 7d).

Overall, these results indicate that in presence of vinblastine, cortical neurons overexpressing either WT or P301S tau develop signs of microtubule severing and axonal damage including formation of filopodia (WT and P301S tau) and neuritic swellings (P301S tau). These effects are significantly decreased in neurons overexpressing 2P tau. The insertion of β-sheet breaking proline residues in regions flanking the second microtubule-binding repeat enhances the ability of tau to stabilize microtubules, most likely by increasing the ability of tau to compete with vinblastine for microtubule binding.

## Discussion

We compared the pathogenic effects of three variants of human tau with point mutations in the microtubule-binding domain that control the propensity of the protein to acquire a β-sheet conformation. Following ICV injection of AAV-tau in the mouse forebrain, we showed motor deficits in the rotarod task caused by tau overexpression. The pro-aggregant P301S tau mutant led to motor impairments that worsened over time. The WT form of tau also induced behavioral deficits, which however did not significantly progress. In contrast, tau overexpression had no effects on motor behavior when β-sheet breaking proline residues were introduced in the microtubule-binding region.

To address the possible mechanisms underlying tau-induced pathology, we determined the main tau species produced in each condition. P301S tau leads to the progressive accumulation of misfolded forms, as well as tau multimers, which correlates with the progression of motor deficits. In contrast, WT tau overexpression produces only low levels of aggregated tau. However, compared to P301S tau, the protein appeared to be more heavily phosphorylated at several specific residues, such as Ser 396, Ser 202/Thr 205, Thr 181 and Thr 231. With respect to WT tau, the 2P variant does has similar abundance of misfolded tau species. Instead, it is characterized by reduced the levels of tau phosphorylation. Consistent with its low levels of aggregation and phosphorylation, 2P tau promotes the stabilization of microtubules both *in vivo* and in cultures of cortical neurons exposed to vinblastine, which suggests that this form of tau is more prone to interact with microtubules.

The pathologic deposition of tau filaments mainly composed of hyperphosphorylated forms of the protein appears to correlate with the symptoms observed in FTDP and Alzheimer’s disease^6–8^. In the molecular processes leading to the tau pathology, the dissociation of the tau protein from the microtubules appears as a primary event, whereas tau hyperphosphorylation may prevent the re-association of the protein with the microtubule network. The amount of tau, with respect to the available sites for microtubule binding, determines the level of free tau protein in the cytosol, which is the starting point towards the formation of pathological tau, either hyperphosphorylated or misfolded into PHF, or both. Therefore, tau overexpression appears as a rational approach to promote the deposition of different forms of tau and assay how they contribute to pathology.

Here, tau was overexpressed following injection of AAV2/6 vectors in the lateral ventricles of neonatal mice. The main advantage of this approach is to induce widespread expression of the protein throughout the mouse forebrain, which is most prominently, but not exclusively observed in the long-projection pyramidal neurons located in the cortex and hippocampus. It is therefore possible to directly compare the pathogenic effects of various forms of tau, when overexpressed at similar levels. Remarkably, this model system leads to a prominent pathology, characterized by tau hyperphosphorylation and misfolding, which is already observed after a few weeks. In the normal brain, the tau protein appears to be transiently hyperphosphorylated during the early postnatal period, although the pattern of tau isoforms expressed during this period is different from the adult brain^32–35^. The overexpressed 4R0N tau isoform might as well be subject to hyperphosphorylation during the early postnatal period, initiating pathological changes that may persist or further progress during adulthood.

**Figure 8:**
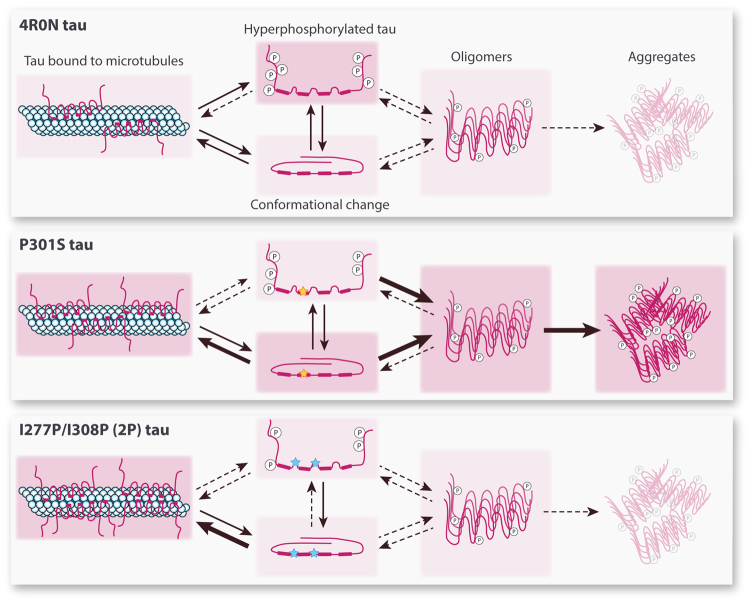
Motifs in the tau microtubule-binding domain affect the development of the tau pathology *in vivo*. Schematic representation of the pathology observed in the mouse forebrain following overexpression of each tau variant. Background colors represent the relative abundance and arrows indicate possible transitions between the different states.

The pathology observed in the adult mice overexpressing each of the three variants of the 4R0N tau protein is summarized in Fig. 8. Remarkably, hyperphosphorylation is most prevalent on the WT form of tau, and appears to be reduced when proline residues are introduced in the microtubule-binding region of the tau protein (2P tau), or in presence of the pathogenic P301S mutation. In particular, the residues Ser396 (PHF1 epitope), Ser202/Thr 205 (AT8 epitope), Thr 181 (the most frequent phosphorylation site measured in clinical AD samples) and Thr 231 are more heavily phosphorylated on WT tau than on P301S and 2P tau. Notably, phosphorylation of Thr 231 can promote the dissociation of the tau protein form microtubules, via conformational changes induced by *trans*-to-*cis* isomerization^36^. P301S and 2P mutations in the microtubule-binding region reduce the propensity of the protein to become hyperphosphorylated and are therefore likely to facilitate tau interaction with microtubules, as suggested by the effect of 2P and P301S tau on microtubule bundling *in vivo*.

Via a β-sheet breaker effect, the 2P modification reduces the propensity of the ΔK280 pro-aggregation variant to accumulate in the sarkosyl-insoluble fraction^37^. The proline residues therefore decrease the toxicity of 2P ΔK280 tau overexpressed in transgenic mice. In the context of WT 4R0N tau, which poorly aggregates in physiological conditions, the 2P modification does not have any apparent effect on the level of MC1-positive or insoluble tau. In the absence of any pro-aggregation mutation, the amount of tau present in these fractions remains low and may not solely depend on β-sheet formation. However, our results support the possibility of a higher interaction of 2P tau with microtubules, possibly facilitated by its low phosphorylation status and/or the presence of these proline residues in key regions of the microtubule-binding domain. This is supported by the observation made in primary cultures of cortical neurons that 2P tau prevents the formation of filopodia in neurites exposed to vinblastine, a microtubule-destabilizing agent. Furthermore, this result suggests that 2P tau may compete more effectively than WT tau with vinblastine, to occupy the tau-binding site at the interface between α- and β-tubulin heterodimers^31^. Therefore, the conformation of the microtubule-binding domain might be critical for the interaction of tau with microtubules^38^.

Remarkably, the behavioral impairments observed in mice overexpressing WT tau are significantly reduced when two proline residues are introduced near the second repeat in the microtubule-binding region. Compared to 2P tau, the enhanced toxic effects of WT tau correlate with higher levels of tau phosphorylation on several residues. Indeed, it has been already demonstrated that the accumulation of hyperphosphorylated forms of tau, most likely dissociated from microtubules, can have detrimental effects in neurons^39,40^. However, these toxic effects are unlikely to be caused by aggregated tau, which remains at low levels in mice overexpressing WT 4R0N tau. Similarly, a mouse model overexpressing 4R1N tau displays cognitive and motor deficits in the absence of any neurofibrillary degeneration, suggesting that phospho-tau and pre-tangle species can cause neuronal dysfunction^22^. In the *Drosophila*, overexpression of 3R0N tau can destabilize microtubules via the formation of soluble hyperphosphorylated species that sequester endogenous tau^11^. Here, we have not observed any significant loss of axonal microtubules in the cortex following AAV-WT injection (see Fig. 6). It is therefore plausible that other mechanisms are implicated, such as disrupted mitochondrial transport, calcium dyshomeostasis and synaptic dysfunction, that may contribute to the induced behavioral deficits^41^.

Tau carrying the FTDP-17-associated P301S mutation has clearly different properties. In contrast to WT tau, the total level of the P301S mutant protein increases over time, indicating an effect of the mutation on tau turnover, possibly via accumulation of insoluble forms of the protein with a longer half-life^42^. Indeed, P301S tau displays enhanced propensity to aggregate, as demonstrated by the constant accumulation of MC1-immunoreactive tau species, and the formation of tau multimers. However, P301S tau appears to be proportionally less phosphorylated than WT tau. The only exception is the increased immunoreactivity for AT100, a phosphorylated tau epitope present at late stages of aggregation characterized by the presence of PHF. Remarkably, and in contrast to WT tau, the behavioral deficits induced by overexpression of P301S tau clearly progress as a function of age. This effect correlates with the increase in the amount of misfolded aggregated tau. The pathogenic effects of P301S tau are also underlined by the axonal damage observed *in vivo* (Fig. 4c), and by the neuritic swellings induced in cortical neurons exposed to vinblastine (Fig. 7d).

Overall, these results show that motifs in the microtubule-binding domain of the tau protein control the pathogenic changes caused by overexpression of human tau *in vivo*. By facilitating the transition towards a β-sheet conformation, the P301S mutation associated with FTDP-17 dramatically enhances tau misfolding and aggregation, despite low levels of phosphorylation, and leads to a rapidly progressing pathology. In contrast, WT 4R0N tau is more extensively hyperphosphorylated. However, the protein does not accumulate into highly aggregated forms, and the behavioral effects of the induced pathology do not progress over time. Nevertheless, it is possible to prevent these behavioral defects by inserting β-sheet-breaking proline residues in the microtubule-binding domain. This modification of the tau protein enhances interaction with microtubules and prevents hyperphosphorylation on the residues that are abundantly phosphorylated in WT tau. This region of the tau protein has therefore a critical role both in the phosphorylation and aggregation of the tau protein, and might be centrally implicated in the pathogenic mechanisms that confer toxic properties to the tau protein.

## Materials and Methods

### Preparation of AAV plasmids

All shuttle AAV plasmids encoding each of the human tau variants were derived from the pAAV-PGK-MCS-WPRE backbone (modified from pAAV-CMV-MCS provided by Stratagene) following standard cloning procedures. The cDNA fragments encoding human 4R0N tau and the P301S mutant were kindly provided by Dr M.G. Spillantini (Centre for Brain Repair, Cambridge University). The human 2P mutant tau (2P tau) was generated by introducing missense mutations encoding the I277P and I308P amino acid substitutions in the 4R0N tau cDNA, using the QuikChange II Site-Directed Mutagenesis Kit (Agilent Technologies). The control vector is derived from the same backbone and encodes the maxFP fluorescent protein.

### Production of AAV vectors

Vector production and titration were performed as previously described^43^. Briefly, each pAAV plasmid was co-transfected with the pDP6 helper plasmid in HEK293-AAV cells (Agilent Technologies). Transfected cells were lysed 48 h later by freeze-thaw cycles. Recovered viral particles were sequentially purified on iodixanol density gradients and heparin affinity columns. Infectivity titers were determined according to the amount of TU measured by real-time PCR, at 48 h after infection of HEK293T cells. The titers obtained for each AAV2/6 vector suspension were: 1.88E + 10 TU/ml, 1.87E + 10 TU/ml and 6.75E + 10 TU/ml for WT 4R0N tau (3 batches); 5.79E + 10 TU/ml and 5.24E + 10 TU/ml for P301S mutant 4R0N tau (2 batches); 2.43E + 10 TU/ml for 2P mutant 4R0N tau.

### Animals

Mice were housed in a room with controlled temperature and maintained in a 12:12 h light:dark cycle, with *ad libitum* access to water and food. All experiments were performed in accordance with Swiss legislation and the European Community Council directive (86/609/EEC) for the care and use of laboratory animals and were approved by the Veterinarian Office of the canton of Vaud and a local ethics committee. Timed pregnant C57BL6/J mice were ordered from Charles River Laboratories (France).

### Vector administration

Newborn mouse pups (C57BL6/J, males and females) were isolated from the home cage at postnatal day 3, kept on a heating pad and anesthetized with intraperitoneal injection of a cocktail containing 0.67 mg/ml medetomidine (Dorbene; Dr. Graeub AG) and 1.7 mg/ml midazolam (Dormicum; Roche) in a total volume of 3.7 μl. The pups were placed on a plastic form mounted on a stereotactic frame and which was especially designed to maintain their head in a fixed position. Simultaneous bilateral intracerebroventricular (ICV) injections were performed using 30 G sharp needles connected to a KDS310 nanopump (KD Scientific). A total dose of 1E + 7 TU of AAV2/6 vector suspension, in a maximal volume of 1.5 µl, was injected in each lateral ventricle at a speed of 1.5 μl/min. Mice receiving virus were randomized during injection day in order to have all groups represented in a given litter. Animals were returned to their home cage after anesthesia reversal with a subcutaneous injection of 5 μl of 1 mg/ml atipamezol (Alzane; Dr. Graeub AG).

### Motor behavior assessment

For behavioral analysis, experimental groups were composed of 12 to 15 sex-matched. General motor behavior was assessed using an open field task. A white square box divided in four compartments (50 × 50 × 37 cm) was used to monitor the spontaneous activity of four mice simultaneously. Under dim and dispersed light conditions, each mouse was placed in the center of the arena and allowed to move freely for 10 min. Various parameters including the total distance moved were analyzed using an Ethovision XT (Noldus) tracking system.

Motor performance was assessed in the rotarod test using a fixed speed protocol. On day 1, mice had two 60-sec training sessions at low speed (10–20 rpm) on the rotarod (Ugo Basile). On day 2, all mice were tested in random order, with two 60-sec trials at various increasing speeds (20, 25, 30 and 35 rpm). The time until the mouse fall down from the rotating rod was measured until the end of the trial (maximal time is 60 sec). The final score for each mouse was the average of two measurements at a speed of 35 rpm. For all motor behavior tests the investigator was blinded to the group allocation of each mouse.

### Tissue processing and histological analysis

Animals were sacrificed with an overdose of pentobarbital and perfused transcardially with heparinized PBS. After opening the skull, the brains were carefully dissected on ice. One hemisphere was kept frozen at −80 °C for later biochemical analysis. The other hemisphere was fixed in 4% paraformaldehyde for 4 h and transferred to 25% sucrose for histological processing. 25 μm-thick sagittal cryosections were collected on Superfrost plus slides (Thermo Fisher Scientific) and stored at −80 °C until further use. For the thioflavin S staining, frozen brain sections were incubated in a solution of 0.01% thioflavin S (Sigma Aldrich) in distilled water for 8 min at RT, washed twice in 50% ethanol for 5 min, and twice in PBS for 10 min. For Gallyas staining, frozen brain sections were treated following a modified Gallyas-silver method. Slides were mounted in 80% glycerol and images of brain sections were taken on a Leica DM5500 microscope (Leica).

### Immunohistochemistry

For the detection of various forms of tau, the following primary antibodies were used: HT7 human specific anti-tau (Thermo Fisher Scientific, MN1000), anti-phosphorylated tau AT8 (pSer202 and pThr205, Thermo Fisher Scientific, MN1020) and PHF1 (pSer396 and pSer404, kindly provided by Dr Peter Davies), anti-PHF tau MC1 (kindly provided by Dr Peter Davies) and AT100 (Thermo Fisher Scientific, MN1060).

After rehydrating the sections in PBS for 15 min at RT, endogenous peroxidases were quenched in 1/1000 phenylhydrazine (Sigma Aldrich) in PBS at 37 °C for 1 h. Sections were then washed 3 times in PBS for 10 min. The blocking of non-specific binding sites was performed by incubating the slides in 5% normal goat serum (Jackson Immunoresearch), 3% bovine serum albumin (Sigma Aldrich) and 0.1% Triton X-100 (Sigma Aldrich) in PBS for 2 h at RT. Primary antibodies were diluted at 1/1000 in blocking buffer and applied overnight at 4 °C. After 3 washes in PBS, a biotinylated goat anti-mouse (Vector Laboratories) secondary antibody diluted 1/200 in PBS was applied for 3 h at RT. The avidin/biotin Vectastain ABC Elite kit (Vector Laboratories) was used for peroxidase detection by Metal Enhanced DAB Substrate Kit (Thermo Fisher Scientific), according to manufacturer’s instructions. Slides were then washed in PBS and mounted in Eukitt mounting medium (Sigma Aldrich). Images of brain sections were taken on a Leica DM5500 microscope (Leica).

### Total brain homogenates

Brains were homogenized on ice using a glass potter in a 9-fold volume of homogenization buffer: 25 mM Tris-HCl pH 7.4, 150 mM NaCl, 1 mM EDTA, 1 mM EGTA containing phosphatase inhibitors (30 mM NaF, 0.2 mM Na_3_VO_4_, 1 nM Okadaic acid, 1 mM PMSF, 5 mM Na_4_P_2_O_7_) and a cocktail of protease inhibitors (Roche). Protein concentration was measured using Micro BCA Protein assay kit (Thermo Fisher Scientific). Protein samples were aliquoted and stored at −80 °C.

For sarcosyl extraction 50 µl of brain homogenates were mixed with 85 µl of homogenization buffer (135 µl final volume). 15 µl of 100% sucrose were added to each sample to then centrifuge at 20,000 g for 20 min at 4 °C. The supernatant was collected and sarcosyl 20% was added to each sample to reach 1% final concentration. Samples were incubated under agitation for 1 h at RT and centrifuged at 100,000 g for 1 h at 4 °C. The collected supernatant corresponded to the sarcosyl soluble fraction. The pellet obtained, called sarcosyl insoluble fraction, was resuspended in 35 µl of 50 mM Tris-Cl pH 7.4.

### Western blotting

Ten μg of proteins were loaded in each lane of a 10% Bis-Tris precast gel (NuPAGE, Life technologies) and run in NuPAGE 1x MOPS SDS running buffer (Life Technologies). After migration of the samples, the proteins were transferred on a PVDF membrane (0.45 µm) (Millipore). Membranes were blocked for 1 h in Odyssey blocking buffer, incubated overnight with primary antibodies (mouse TAU-13 or rabbit anti-actin (Abcam), washed three times with PBS 0.1% Tween-20, incubated 1 h at RT with secondary anti-Mouse-IRDye CW-800 or anti-Rabbit-IRDye CW-680 antibodies (Odyssey). The membranes were finally scanned using LICOR (Odyssey Infrared Imager) and quantification performed using the LICOR software.

### AlphaLisa assays

For the quantification of various tau species in brain homogenates, different AlphaLisa assays were developed according to manufacturer instructions (Perkin Elmer). Acceptor beads were conjugated to either the HT7 anti-human tau antibody (Thermo Fisher Scientific, MN1000) or the TAU-13 anti-human tau (Abcam, B11E8). Detection antibodies pSer202/pThr205 (AT8, Thermo Fisher Scientific, MN1020), pThr181 (AT270, Thermo Fisher Scientific, MN1050), pThr231 (AT180, Thermo Fisher Scientific, MN1040), human tau (HT7, Thermo Fisher Scientific, MN1000), pThr212 (Santa Cruz Biotechnology), MC1 (kindly provided by Prof. Peter Davies), pSer409 and pSer396 (AC Immune) were either purchased with biotin tag or conjugated to biotin using EZ-Link™ Sulfo-NHS-Biotin (Thermo Fischer Scientific). Brain total homogenates were pre-diluted in PBS to obtain a 5 µg/µl stock concentration. Dilutions of all reagents were made with Alpha Assay buffer (PerkinElmer). Reagents were added in a 384-well white OptiPlate (PerkinElmer) to a final volume of 50 µl. 5 µl of total brain homogenate (final concentration in an assay: 0.5 µg/µl), 10 µl of biotinylated detection antibody (final concentration: 10 nM), 10 µL of tau acceptor beads conjugate (final bead concentration: 2.5 µg/ml) were incubated 1 h at RT, before the addition of 25 µl of donor beads (final bead concentration: 25 µg/ml) and a further incubation at RT for 30 min (protected from light). The plate reading was performed using EnSpire Alpha instrument and analysis using EnSpire Workstation version 3.00.

### Electron microscopy

Anaesthetized animals were perfused with a buffered solution of glutaraldehyde (2.5%) and paraformaldehyde (2%), and then vibratome sectioned at 80 µm thickness cut through the mouse cortex, close to the site of injection. Selected sections were then further fixed with 1.5% potassium ferrocyanide and 1% osmium tetroxide, followed by 1% osmium alone, and then uranyl acetate. After dehydration in a series of ascending concentrations of ethanol, they were embedded in durcupan resin and hardened at 65 °C for 24 h. Thin sections at 50 nm thickness were cut through the region of interest, stained with lead citrate and uranyl acetate, and imaged in a transmission electron microscope at 80 kV (Tecnai Spirit, FEI Company). To quantify microtubule number and density, 30 axonal cross-sections per sample were randomly analyzed in the mouse cortex, using the TrakEM2 plugin (Fiji)^44^. For each axon, the number of microtubules was counted and divided by the measured cross-section area to determine microtubule density.

### Primary cortical neurons and vinblastine treatments

Frontal cortex tissue was dissected from E16 C57BL/6 J mouse embryos following standard methods. We performed AAV2/6 infections at DIV1 to induce transgene expression using a viral dose of 2.5E + 5 TU per well. For this experiment, we used a bicistronic vector construct co-expressing a nuclear GFP to identify the transduced neurons (AAV2/6-PGK-Tau:CMV-nlsGFP). To determine the effects of human tau variants in presence of vinblastine, AAV2/6-infected neurons were incubated at DIV4 during 24 h or 7 days with 1 nM vinblastine and fixed with 4% PFA at DIV5 or DIV11. AAV2/6-transduced mouse cortical neurons overexpressing human tau were immunostained using the HT7 (Thermo Fisher Scientific, MN1000) and GFP (Thermo Fisher Scientific, A-11122) antibodies. For morphological analysis, all conditions were performed in triplicate.

For the quantification of filopodia, we took from each coverslip five high-magnification images (40x) where individual neurites could be visualized. To ensure comparable abundance of human tau for each condition, we assessed the morphology of neurites with similar intensity of the human tau immunostaining. A total of 15 images were analyzed for each condition in a blind manner. The number of filopodia present per 100 µm of neurite length was measured using ImageJ.

To quantify neuritic swellings, five low-magnification images (20x) of individual neurites were taken from each coverslip, with similar intensity of the human tau immunostaining. A total of 15 low-magnification images were analyzed for each condition in a blind manner. For each image, the number of swellings present per 100 µm of neurite length was measured using ImageJ.

### Statistical analysis

All statistical analyses were performed using the Statistica V.6 software and graphs were prepared using Microsoft Excel. Student’s t test, Kruskal-Wallis test, and two-way ANOVA followed by different post-hoc tests were used to compare different groups. Parametric Student’s or ANOVA tests were applied to normally distributed data sets with equal variance. Since this study used a new animal model, we did not have any preliminary data set to decide the sample size based on a statistical method. Hence, we have estimated the sample size based on literature and our previous experience with similar models. No animals were excluded from the study for the data analysis. For all graphs data represent mean ± SEM.

### Data availability

Authors agree that all relevant data can be made available in case of need.

## Electronic supplementary material

Supplementary Information
