# Supplementary material for: Motifs in the tau protein that control binding to microtubules and aggregation determine pathological effects
Source: Sci Rep. 2017 Oct 19;7:13556. doi: 10.1038/s41598-017-13786-2 (PMC5648870; doi:10.1038/s41598-017-13786-2)
Supplement: Supplementary file 1 — Supplementary Information [file 41598_2017_13786_MOESM1_ESM.pdf]

Motifs in the tau protein that control binding to microtubules and aggregation determine pathological effects

Aurélien Lathuilière[1#], Pamela Valdés[1#], Stéphanie Papin[2,4], Matthias Cacquevel[1], Catherine Maclachlan[3], Graham W. Knott[3], Andreas Muhs[2], Paolo Paganetti[2,4], Bernard L. Schneider[1*]

Supplementary information

Supplementary Figure S1

Supplementary Figure S2

Supplementary Figure S1

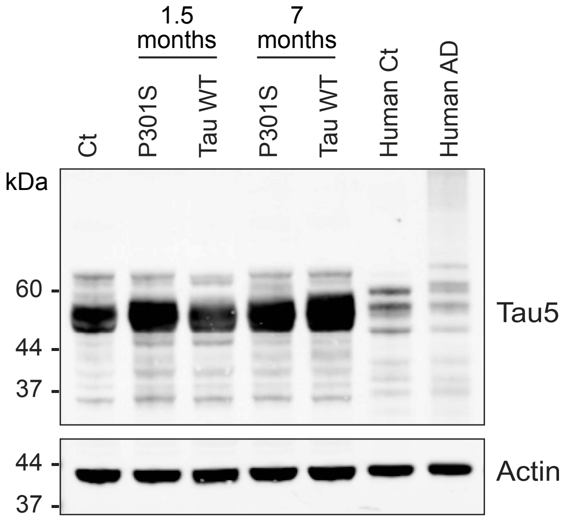

Supplementary Figure S1. Total tau expression in the forebrain of mice injected with AAV-tau

The amount of total tau protein (mouse and human) is assessed by western blot using the Tau5 antibody in mouse forebrain protein homogenates at 1.5 and 7 months after vector injection. Ct: control AAV-maxFP injection. Protein extracts from human brain cortex from a control (Human Ct) and Alzheimer's patient (Human AD) are shown in the last two lanes for comparison. Actin is shown as loading control.

Supplementary Figure S2

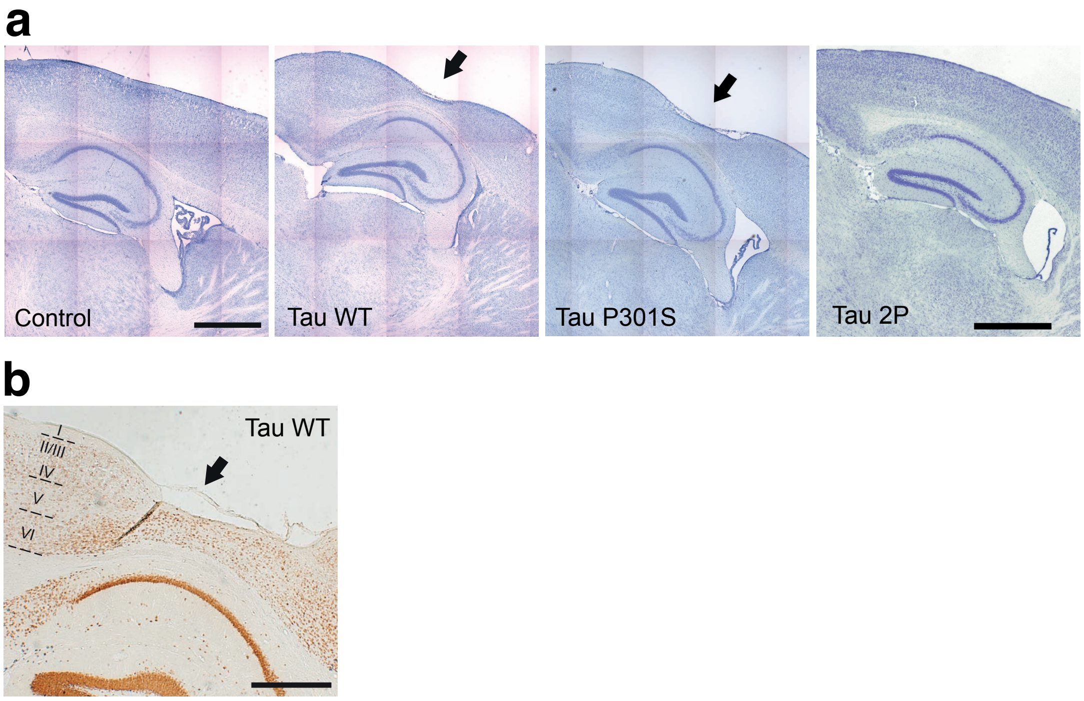

Supplementary Figure S2. Human tau overexpression triggers neurodegeneration in mouse cortex

a) Representative cresyl violet staining in sagittal brain sections, 3 months after AAV injection. Arrows indicate an evident thinning of the cortex observed in the mice injected with AAV-WT and AAV-P301S. Scale bar: 1 mm. **b**) Representative NeuN immunolabeling in a sagittal brain section of an AAV-WT injected mouse. Cortical layers are indicated by roman numerals. Note the extensive degeneration of the most superficial cortical layers. Scale bar: 500 μm.
